# Best Practice Recommendations: User Acceptance Testing for Systems Designed to Collect Clinical Outcome Assessment Data Electronically

**DOI:** 10.1007/s43441-021-00363-z

**Published:** 2022-03-01

**Authors:** Sarah Gordon, Jennifer Crager, Cindy Howry, Alexandra I. Barsdorf, Jeff Cohen, Mabel Crescioni, Bela Dahya, Patricia Delong, Christian Knaus, David S. Reasner, Susan Vallow, Katherine Zarzar, Sonya Eremenco, the Electronic Patient-Reported (ePRO) Consortium, PRO Consortium

**Affiliations:** 1 PPD, Wilmington, NC USA; 2 PPD, Lubbock, TX USA; 3.assisTek, Austin, TX USA; 4 Clinical Outcomes Solutions, Chicago, IL USA; 5 Janssen Research & Development, Raritan, NJ USA; 6 Hemophilia Federation of America, Washington, DC, USA; 7 Clinical Ink, Winston-Salem, NC USA; 8 Janssen Global Services, LLC, Raritan, NJ USA; 9 Science 37, Los Angeles, CA USA; 10 Imbria Pharmaceuticals, Boston, MA USA; 11 Novartis Oncology, East Hanover, NJ USA; 12 Genentech, Inc., A Member of the Roche Group, South San Francisco, CA USA; 13 Critical Path Institute, Tucson, AZ USA

**Keywords:** eCOA, ePRO, Project lifecycle, SDLC, UAT, User acceptance testing

## Abstract

Implementing clinical outcome assessments electronically in clinical studies requires the sponsor and electronic clinical outcome assessment (eCOA) provider to work closely together to implement study-specific requirements and ensure consensus-defined best practices are followed. One of the most important steps is for sponsors to conduct user acceptance testing (UAT) using an eCOA system developed by the eCOA provider. UAT provides the clinical study team including sponsor or designee an opportunity to evaluate actual software performance and ensure that the sponsor’s intended requirements were communicated clearly and accurately translated into the system design, and that the system conforms to a sponsor-approved requirements document based on the study protocol. The components of an eCOA system, such as the study-specific application, customization features, study portal, and custom data transfers should be tested during UAT. While the provider will perform their own system validation, the sponsor or designee should also perform their due diligence by conducting UAT. A clear UAT plan including the necessary documentation may be requested by regulatory authorities depending on the country. This paper provides the electronic patient-reported outcome (ePRO) Consortium’s and patient-reported outcome (PRO) Consortium’s best practice recommendations for clinical study sponsors or their designee for conducting UAT with support from eCOA providers to ensure data quality and enhance operational efficiency of the eCOA system. Following these best practice recommendations and completing UAT in its entirety will support a high quality eCOA system and ensure more reliable and complete data are collected, which are essential to the success of the study.

## Introduction

**Fig. 1:**
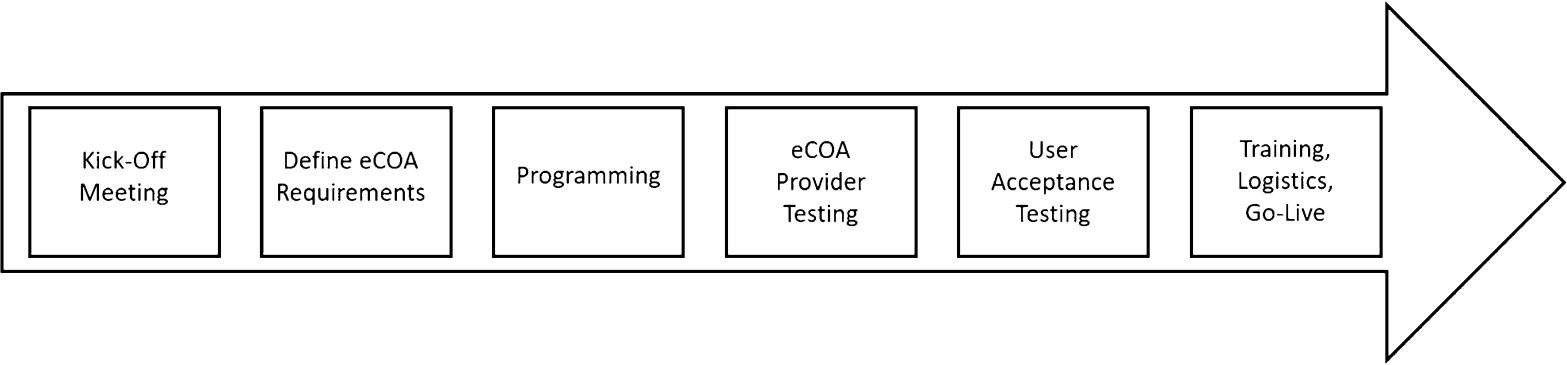
Typical eCOA implementation process

The collection of clinical outcome assessments electronically in clinical studies involves a process that requires clinical study sponsors and electronic clinical outcome assessment (eCOA) providers to work closely together to implement study-specific requirements, incorporate best practices, and ensure successful data collection to generate evidence for regulators and other stakeholders including payers and health technology assessment bodies. There are multiple steps in the system development process (Fig. 1), most of which have been discussed in the literature [1, 2] and regulatory guidance [3–5]. However, one of the most important steps in this process, user acceptance testing (UAT), which aims to ensure that an electronic system functions according to agreed-upon requirements (e.g., business requirements document based on the study protocol), deserves increased attention. Therefore, Critical Path Institute’s electronic patient-reported outcome (ePRO) Consortium and patient-reported outcome (PRO) Consortium have developed UAT best practice recommendations for clinical study sponsors or their designee for conducting UAT with support from eCOA providers to ensure data quality and enhance operational efficiency of the eCOA system. Utilizing these best practices should improve the reliability or precision of clinical outcome assessment (COA) data collected electronically in clinical studies to support product registration.

The United States Food and Drug Administration’s (FDA’s) “General Principles of Software Validation; Final Guidance for Industry and FDA Staff” outlines regulatory expectations for software validation [3]. This guidance states that terms such as beta test, site validation, user acceptance test, installation verification, and installation testing have all been used to describe user site testing which encompasses any other testing that takes place outside of the developer’s controlled environment. For purposes of this paper, the term “UAT” will be referenced and “user” will refer to sponsor staff (or designee) who serve as substitutes to trial participants for the participant-facing components of the eCOA system. The FDA general principles go on to say that “User site testing should follow a pre-defined written plan with a formal summary of testing and a record of formal acceptance. Documented evidence of all testing procedures, test input data, and test results should be retained” [3, p. 27]. These statements in the guidance indicate that a user testing process itself as well as documentation are both best practices in software development as well as regulatory expectations.

In 2013, the International Society for Pharmacoeconomics and Outcomes Research (ISPOR) ePRO Systems Validation Task Force defined UAT as “the process by which the clinical trial team determines whether the system meets expectations and performs according to the system requirements documentation” [2, p. 486]. In this same report, the task force also indicated that UAT should not be “a complete revalidation effort conducted by the sponsoring clinical trial team” [2, p. 486]. but, rather, a “focused, risk-based approach to testing that allows the clinical trial team to determine whether the system complies with the key system requirements (which ultimately reflect the protocol)” [2, p. 486]. Because differentiating between the specific activities recommended for UAT and those activities conducted during system validation can be confusing, these best practice recommendations were developed to clarify those activities and considerations that should be accounted for during UAT by the sponsor or designee. A separate process called usability testing involves participants and evaluates their ability to use the system as intended for the purposes of the study, which is outside the scope of this paper. See Coons et al. [1] and Eremenco et al. [6] for more information on usability testing, and FDA’s *Discussion Document for Patient-Focused Drug Development Public 2 Workshop on Guidance 3* [7] which discusses both usability testing and UAT.

The concept of UAT comes from the software development lifecycle (SDLC) and is intended to test how the system would perform in circumstances similar to those in which the system will eventually be used. In clinical studies where electronic systems are being used to collect COA data, UAT provides the clinical study team, including sponsor and/or contract research organization (CRO) representatives, an opportunity to evaluate actual system performance and ensure that the sponsor’s intended requirements were communicated clearly and accurately translated into the system design, and that the system conforms to a sponsor-approved requirements document.

System requirements should be thoroughly tested by the eCOA provider prior to UAT in conformance with the SDLC process implemented by the eCOA provider. The eCOA provider project manager will notify the sponsor and/or designee when the vendor testing process is completed so that UAT may proceed. This step followed by the eCOA provider allows the focus of UAT to remain on a common understanding of the requirements with the actual system in hand, as well as identifying and correcting issues proactively that study team, site, and study participant users might experience once the system is deployed. UAT takes place toward the end of the eCOA implementation process (Fig. 1), occurring after the study-specific system requirements have been documented by the eCOA provider and approved by the study sponsor, and the system is built and tested by the eCOA provider’s in-house testing team. UAT must be completed prior to launching the technology for the study.

## Components of an eCOA System

eCOA systems are built differently by each eCOA provider but typically have the same core components. Table 1 provides the suggested guidelines for testing these components in terms of when formal testing using UAT scripts is recommended as a best practice as opposed to cases where ad hoc testing may be sufficient. Details on the development of UAT scripts are provided in the UAT Documentation section of this paper. eCOA systems can be deployed on provisioned devices. If the study is utilizing a provisioned device model, the eCOA provider will distribute devices to each tester. eCOA systems can also contain components that are application-based such as those developed for Bring Your Own Device (BYOD) studies, where a variety of devices (including different makes and models) should be included in the formal UAT to ensure consistency between device types. If a study is utilizing a BYOD setup, the eCOA provider is required to provide the testers with minimum operating system and device requirements (e.g., Android/iOS operating system versions, internet browser, screen size). If feasible to be done at the time of the eCOA UAT, testing of any integrated devices (e.g., glucometers, sensor patches) or systems (e.g., IRT, EDC), should also be included within the component testing of UAT. For purposes of this paper, best practices for testing integrated devices or systems will not be covered.

**Table 1:** eCOA system components and testing guideline

Component	Description	Guidelines for testing
Core system	The core system is the computer system that is the base of the eCOA platform (i.e., the provider’s proprietary system). This core system is built using a software development lifecycle which includes programming and testing by the eCOA provider. The system is supported with full validation documentation and is versioned when changes are made. The core system is the starting point before any study-specific programming is completed and will usually include the various eCOA modalities such as single user application (subject) software, multi-user software (site-based), and system portal. Examples of what the core system would include: site setup and login, subject setup and login, activation, deactivation, discontinuation, reactivation, data transmission. This is not an exhaustive list but provides examples of what is considered core. The core system may also include backup options such as a web-based application, which would also need to be tested during UAT	Prior to UAT, eCOA providers will use a software development process for building and testing their core software. The eCOA provider’s testing includes diligent validation and documentation to ensure the system is functioning per its specifications. Although this is the case, it is still strongly advised that the sponsor or designee conducting UAT test any core functionality that impacts the study-specific software and/or the user experience within the study formally using UAT scripts (see "UAT Documentation" section). An example would be verifying the login to the system. Although the eCOA provider has diligently tested this functionality, UAT should include steps to log into the system and ensure the user experience is correct per user requirements. Supplemental ad hoc testing can also occur for any functionality that is available. An example of ad hoc testing would be to portray the role of a subject and go through three days of diary entries entering values to instigate error messages
Study-specific application	The study-specific application includes programming completed by the eCOA provider to incorporate the study-specific software requirements. This layer may include questionnaires, study-specific training, visit schedules, and study-specific alarms, notifications, and rules. eCOA providers will most likely use a design tool including various functionality widgets to design the study-specific content. Examples of the study-specific application would include: PRO measure-specific programming such as skip logic, ordering of assessments, schedule of activities, calculations such as inclusion criteria. This is not a complete list but provides examples of what is considered the study-specific application	During UAT, it is a best practice to conduct a formal testing process using test scripts to guide the testing and document test results (see "UAT Documentation" section) for the application layer of an eCOA system. As this software is designed specifically for a study, all functionality must be thoroughly tested. It is suggested that test scripts note step-by-step instructions to confirm functionality of every function designed for the study. Examples would include questionnaire screen content, skip logic, visit schedule functionality, and alarms. The test scripts should also include scenario (user stories) testing and may include real world testing (e.g., taking a device home to enter events in real time). If different functionality is in place for different days of the study, sample days of each variation should be tested
Custom software	If there is specific software needed for the project where the functionality is not in place in either the core system or within the application tool/designer, the eCOA provider will use custom programming to alter the study-specific application to accommodate the software requirement such as custom alerts, eligibility calculations, or visit schedule	Unlike the application layer where there is a tool used to create the software, custom software is created by a programmer for the one instance of change to the system. Testing using detailed UAT scripts is a best practice for custom software to ensure the software was created and implemented correctly. It should follow the same process described in the Study-Specific Application section
System portal	The system portal is designed to display information for the end user. A core system is typically in place to display the system data and standard reports. A study-specific layer may need to be programmed to accommodate custom reports or blinding elements (e.g., Eligibility Report) or other applications	Similar to testing the core system, the study-specific portal sections should follow the same methodology where anything created for study-specific use should be formally tested using UAT scripts while ad hoc testing can support the other functions of the portal. An example of a required script would be for a custom Eligibility Report or whether certain fields are suppressed for a particular role. An example of ad hoc testing would be to check the enrollment status of a given site
Data exports	The data extracted from the eCOA system. This can be the raw data directly downloaded from the eCOA system or a customized data export programmed by the eCOA provider. The primary purpose of testing the data export is to ensure the outputs of the eCOA entry were submitted and stored correctly in the system	As data are the output of the eCOA system, test cases should be developed using the inputs from the study-specific test cases. Scripts should cover test cases such as verification of all required fields (e.g., date/time, responses). In some cases, custom exports may not be available at the same time as the main study UAT. In these cases, testing of the data export should be completed once the export is available

## eCOA Hosting Environments

A hosting environment is the physical server environment in which the eCOA platform resides. eCOA providers should have multiple hosting environments to support a proper setup. Typically, all development of an eCOA system is done within a Development (or Dev) environment. In the Dev environment, the eCOA provider builds the system per the study requirements and can easily make changes as needed. The Dev environment is sometimes referred to as a sand box as the eCOA provider is able to modify the design without impact to test or live study data.

Once the development of the software application is completed, system/integration testing of the software application is performed by the eCOA provider in a Test environment. After this process is completed by the eCOA provider, UAT should be performed by the sponsor or designee who is provided access to the software application in a separate UAT environment hosted by the eCOA provider.

Once UAT has been completed successfully, with no outstanding issues, and all parties agree that the system is acceptable for study use, the study configuration is moved to the Production environment. The Production environment will collect only live study data. UAT should not be performed in a Production environment under any circumstances, as UAT data could end up in a live system database. In the event that the study requirements change (e.g., due to a protocol amendment) once the system is live, any post-production changes must be made in the Development environment and subsequently tested in the Test environment by the eCOA provider and UAT environment by the sponsor or designee before moving the modified study configuration to the Production environment.

## Roles and Responsibilities

When planning and executing UAT for an eCOA system implemented for a clinical study, there are two main expected stakeholders, which can be categorized on a high level as:

1. Sponsor or designee: the entity for whom the system is built and who funds both the build and clinical study, and who has ultimate accountability for the study overall. Note that a CRO and/or UAT vendor may be engaged to act as a designee of the sponsor to perform UAT.
2. eCOA Provider: the entity who is contracted by the sponsor or CRO to carry out the design, build, and support of the system

These primary stakeholders can delegate or outsource roles and responsibilities to any degree necessary to a third party. It is recommended that the Sponsor (or designee) performing UAT ensures all testers are fully trained in the UAT process. In addition, it is recommended that a range of study team roles be involved in UAT execution, including for example, clinical operations, site monitoring, data management, and biostatistics. It is not a best practice for the eCOA provider’s staff to conduct UAT, as it should be conducted by a separate entity to ensure it is objective. It is important to note that study participants are not included in UAT as a standard practice because of its emphasis on formally testing requirements.

## UAT Stages

Each UAT should go through the basic stages of Planning, Execution, and Follow-Up/Closeout, and all stakeholders should participate in each stage. Table 2 details the ideal level of involvement and responsibilities by stage.

**Table 2:** Stages and stakeholder responsibilities

	Sponsor (CRO, or other 3rd party if outsourced)	eCOA provider
UAT planning	Work with the eCOA provider to define a timeline Identify group of testers (should not include eCOA provider representatives) Develop UAT test plan to define scope Develop UAT test scripts Train users on how to properly execute test scripts	Provide system requirements, manuals and other documentation needed by the sponsor and/or its designee to plan and prepare for UAT Collect details needed to provide system access or ship hardware Develop UAT training to set tester expectations and explain how to use the electronic UAT system Provide UAT Findings Log template to ensure issues are consistently tracked by different testers Provide instructions for time travel or manipulation (the mechanism by which testers can move between different dates and times by adjusting the eCOA device clock), if needed Provide capability for data loading, if applicable
UAT conduct	All testers execute test scripts All testers document findings in test scripts Collect and summarize findings in the log, consolidating those identified by multiple testers Optionally, testing may utilize actual data entered by testers	Hold a UAT Kick-Off and/or training Provide the UAT environment for the UAT of the systems to be evaluated. If available, also include access to backend databases and other tools to enable the accurate assessment of test findings (e.g., study dashboards)
UAT follow-up/closeout	Participate in debrief meeting to discuss findings and impact and agree on action plan, if any Re-test any findings that were raised and fixed Develop UAT Summary Report to document all testing activities including any findings and their resolution Approve UAT, when applicable, thus allowing the eCOA provider to release system for the study	Analyze any findings for impact Lead debrief meeting to discuss findings and impact and agree on action plan, if any Make any requirements and/or system updates as necessary. Re-release for re-test as deemed appropriate Require documentation of sponsor approval to release the system Follow other processes if required by provider’s standard operating procedures (SOPs) Release system to the study production environment following UAT approval

Table 3 outlines primary responsibilities for the tasks necessary to conduct UAT.

**Table 3:** Task ownership matrix

Task	Sponsor or designee	eCOA provider
Determine timeframe for UAT	X	X
Identify testers	X	
Develop UAT test plan	X	
Develop test scripts	X	
UAT systems and access		X
UAT kick-off/system training		X
UAT execution/testing	X	
UAT summary report	X	
UAT debrief meeting		X
Analyze findings		X
UAT approval form	X (if applicable)	X
System release		X

## UAT Conduct

A UAT timeline can vary; however, it is best to plan for at least a 2-week cycle that assumes multiple rounds for UAT including testing as outlined in the test plan and scripts, changes, re-verification, and final approval. UAT timelines are also dependent on the complexity of the study design including the number of treatment arms, assessments, visit schedule and when the system build will be fully validated by the eCOA provider versus the planned date for the system to be launched for the study as UAT is often the rate-limiting step that must be completed to launch the system. The UAT timeline can be extended or shortened depending on these variables and the actual iterations of rounds of testing needed. Regardless of the length of time for UAT, time for testing, changes, validation of changes by the eCOA provider test team, and re-testing by the UAT team needs to be accounted for prior to a system launch. If these steps are not carried out, the potential for issues and reduced system quality increases.

While UAT is being conducted, each tester should document findings within the test script(s) and provide all findings (issues/questions/changes) within a UAT findings log. This log can be in several different formats such as spreadsheets or an electronic UAT system. At the completion of each round of testing, findings should be collated into one log for ease of review by sponsor and/or designee team with duplicate issues removed. Following each round of UAT, a debrief meeting should be held to examine and discuss all findings as a team. It is important for all testers to be represented at the meeting so that each finding can be discussed and clarified as necessary. The team may prioritize UAT findings and decide on a phased implementation based which bugs/errors must be corrected ahead of Go-Live vs. those that can be implemented in a “Post Go-Live release plan.” If this approach is taken, it is critical to get agreement between the sponsor and the eCOA provider along with a communication plan to the study team members. Impact to the Data Management Review Plan and Study Site Monitoring Plans should also be evaluated for impact.

Issues (bugs) or changes identified in the UAT findings log need to be categorized to determine their priority and relevance. Categories may include system issue, application or software bug, design change, enhancement, or script error, all of which may have different names, depending on the eCOA provider, but ultimately these categories help determine the corrective plan of action (if necessary). A system issue is a problem in the software programming that causes the system to function incorrectly, which is a critical finding and should be prioritized over all other findings for correction and re-testing. An application or software bug is an error, flaw or fault in a computer program or system that causes it to produce an incorrect or unexpected result, or to behave in unintended ways. An issue is an instance where the agreed-upon requirements were not met. Design changes are requests for changes to the system but are not error corrections while enhancements are requests for improvements to the system that arise from the UAT. Design changes and/or enhancements should be evaluated by the full team to determine whether the change would improve the performance of the system and/or user experience as well as whether time permits the change to be made within the constraints of the system launch date. Enhancements or changes to the original system design need to be reviewed carefully between the sponsor and the eCOA provider as system design changes create risk and fees may be charged if a change request is deemed out of scope or an expansion of the previously agreed scope. Script errors are mistakes in the script that may lead to erroneous results although the system actually performs correctly. Script errors should be documented and updated in the script template to ensure any future user of the script does not encounter the same problem(s).

While discussing changes resulting from UAT, the original scope of work should always be reviewed and referenced when considering implementing the change. eCOA providers should correct any programming errors found in UAT at no additional cost. If necessary design features not included in the original requirements document are identified as a result of UAT, sponsors are advised to consider the timeline and cost implications of introducing those new features at this late stage. If it is deemed the changes are required prior to launch, the sponsor may need to accept any additional costs or delays to launch, depending on the assumptions built into the original contract. Alternatively, the team may decide that although changes should be made, they are not needed for launch and can be made as a post-production change, after the system is launched into production. The UAT testers and other sponsor representatives should discuss the cost and timeline implications for any options prior to making a final decision about design changes. Involvement of the key stakeholders during the bidding and design process is an ideal way to reduce/limit design changes and expedite processes between the sponsor/CRO and the eCOA provider.

## UAT Documentation

**Fig. 2:**
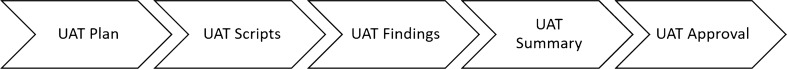
UAT documentation workflow

Proper documentation is imperative to ensure execution of effective testing as shown in Fig. 2 and to meet regulatory expectations. UAT documents should include a UAT test plan, test scripts, findings log, a summary of issues and resolutions (e.g., UAT Summary Report), and lastly, a UAT approval form. The eCOA provider may generate additional documentation such as instructions related to”time travel” (the mechanism by which testers can move between different dates and times by adjusting the eCOA device clock) to assist UAT.

**Fig. 3:**
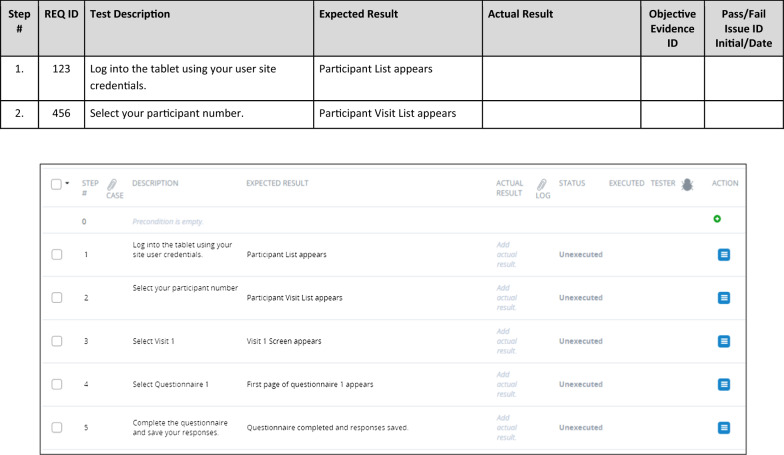
Example of manual and electronic test script

Standard Operating Procedures (SOPs), Working Instructions, and/or guidance documents, and performance metrics for UAT should be developed by the sponsor or designee who is managing UAT to document the requirements for the process and all necessary documentation. UAT SOPs should outline how clinical study teams determine whether the system performs in accordance with the finalized system requirements document. SOPs should define the documents required to complete the UAT, those responsible to perform testing, and when and how UAT is to be performed. Frequency of UAT is also defined in the SOP depending upon initial production releases, updates to correct issues, and/or updates requested by the sponsor. UAT documentation should be made available for audits / inspections and follow Good Clinical Practice as well as appropriate record retention (Fig. 3).

### UAT Test Plan

The UAT test plan is developed by the sponsor or designee and may contain: a purpose, a scope, definitions, references, strategy and approach, assumptions and constraints, risk assessment, UAT team roles and responsibilities, information about the test environment(s), a description of all test cases, scripts, deliverables, the UAT test summary (results), and approvals/signatures. A UAT test plan ensures all parties are aware of the scope and strategy of how requirements will be tested. It will allow the sponsor or designee to review the system per the protocol and the signed system requirements document. As such, it should be considered the first document to be created within the UAT process. The following sections should be considered when creating the Test Plan (Table 4).

**Table 4:** UAT test plan template content

Section	Description
Document objective	Test scenarios/test objectives that will be validated
Referenced documents	List of documents referenced
Definitions	Define acronyms and terms used in test plan; define symbols in applicable diagrams
In-scope	Test scenarios/test objectives that will be tested including software and portal for viewing data
	Core system (as applicable to the study)
	Study-specific application
	Portal
Out of scope	Core system not applicable to the study
Assumptions	All the conditions needed to be able to proceed successfully
UAT schedule	Write test plan and scripts
	Review test plan and scripts
	Setup UAT environment (can be done concurrently while test plan and test scripts are being written)
	Train users on UAT environment
	UAT test execution
	Review test data and findings log
	Update the system if necessary and maintain version control of appropriate documentation
	UAT approval
Roles and responsibilities	Team members are documented
	Determine who is doing what
Functionality	Methodology for testing (See Table 5)
Environment	Defined UAT environment
Tools	Bug tracking tool
	Findings log (used by the UAT testers to input findings)
Risk assessment	Assess and document the risks that the UAT strategy should address
Completion and approval	All issues resolved, unless noted
	Enhancements, if any, discussed
	UAT Approval Form fully executed documenting enhancements, modifications, and any outstanding open issues

Table 5 provides several considerations for testing functionality that is common across eCOA providers. The screen interface may include different controls to navigate from one screen to the next and buttons or graphic controls to select responses to items; these elements are referred to as screen controls. In addition, the Test Plan should include the method for testing custom features for each study.

**Table 5:** Functionality and methodology for testing

Functionality	Methodology for testing
Default screen appearance	When screen is displayed, verify that the screen appearance matches the applicable figure in the system requirements document
	For screens that have different appearance based on the method by which the screen is reached, verify that the screen appearance matches the requirements for each possible path
Screen controls: single-select response	Verify that the system allows the user to select only one response on single-select items (for which only one response is allowed)
	Verify that the user can switch between response selections or de-select an option per the requirements document
	Verify that the system behaves as appropriate based on the row selected
	Verify that the field is enabled/disabled appropriately
Screen controls: icons	Verify icons perform the functions defined in the requirements document
	Verify the appearance of the icons
Buttons and screen flow logic	Verify that when each button is selected, the system displays the message or new screen as appropriate
	Verify that the system does or does not save data based on button selection as appropriate (e.g., Cancel vs. OK)
	Verify system follows each possible logic path by independently producing each condition that initiates the logic
Acceptable field values	Verify that the system presents the appropriate error message when the user attempts to enter a value that is not accepted by the field (negative testing)
	Verify that the system allows all intended values depending on field type (positive testing)
	For fields that accept a range, attempt values at the end of the range and the value immediately past the range (boundary testing)
Measures	Verify correct COA measures are displayed in application according to requirements document and protocol and that content of the COA measures is correct
	Verify correct version of COA measure is displayed, if applicable
Completion windows	Verify measures are available during specified windows of time based on requirements document
	Verify measures are not available during windows of time when they should not be available

### Test Scripts

Test scripts outline each step that a tester will take to test the use cases in the system. Test scripts are designed to be followed step-by-step so that the tester does not have to try to remember how he or she arrived at a given screen. If the step occurs as expected in the script, the tester indicates “pass.” If something happens when the step is carried out that is not as expected, the tester indicates “fail” and provides a reason for failure, with applicable screenshots, if necessary. UAT test scripts will be referenced in the UAT Test Plan. It is best practice that the sponsor or designee write the test scripts and not ask the eCOA provider to provision test scripts. Test scripts should be approved by the appropriate individual within the sponsor or designee prior to UAT being conducted. The approver may vary depending on sponsor UAT process and SOPs. Upon completion of the scripts, the tester should sign (or electronically sign) as well as record the date(s) of script execution.

In some cases, a tester may informally test functionality that is not detailed in the test script, which is referred to as ad hoc testing; for example, this might occur when the actual results of a test step are not the expected results, and ad hoc testing might help identify the root cause of the issue. While such ad hoc testing can be useful in identifying issues, it is considered supplemental and should not be conducted in place of following test scripts. Any issue detected in ad hoc testing should be documented and formally tested in the next round of UAT to document resolution.

Table 6 outlines the aspects that should be documented in each test script section:

**Table 6:** Test script content

Section	Definition
Document history	Capture reason for change with version change, if any
UAT objective(s)	Describe purpose of UAT including what has been tested
Prerequisite	Capture prerequisite to perform UAT
Test script	Capture step-by-step process to perform testing with step description and its expected result in the test script template; Capture response/selection entered by the tester and actual Result during testing with pass/fail result and any comment you might want to add (see Fig. 3)
Comments	General comment regarding test script or its result
Pre-execution script approval	Capture reviewer signature with date for pre-execution approval
Script execution	Capture tester’s name and step executed with signature and location (in case multiple testers are involved) (see Fig. 4) or if electronic system, name may be automatically added
Overall test result	Capture overall pass/fail result of the script that was tested and description of apparent failure along with rationale (see Fig. 4)

**Fig. 4:**
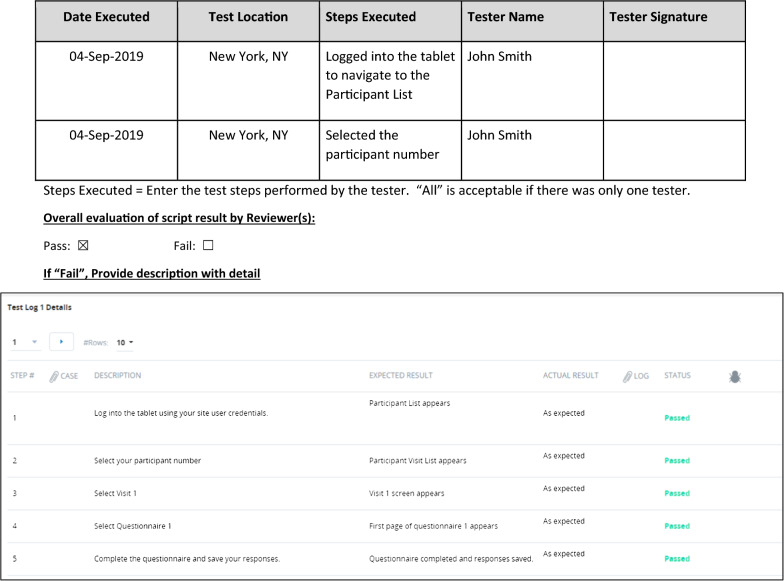
Example of test script execution

If any step in a script “fails” due to an issue with the system, device, or configuration, then the entire test case fails (see Fig. 4). If a test case fails during UAT, the test case should be completed again once the eCOA provider has confirmed that the issue has been resolved. If it is between the sponsor and the eCOA provider that the issue will stay unresolved in the system, then it should be noted in the UAT summary report (results). Otherwise, UAT should not be considered finished until all test cases have been passed by a tester and all issues from the findings log addressed.

If a test case fails due to a script error, retesting of the test case may not be required. The UAT team should identify whether a retest is required for a test case failure due to script error. For example, if a script contains a typographical error or is poorly written but the test case still proves and supports the scope of the test, it is acceptable to amend the script and pass the test case.

Before UAT approval, a UAT summary or report should be created by the UAT testing team (sponsor or designee) summarizing the results of testing including any known issues that will not be corrected in the system before the system is launched into production.

A UAT approval form should be signed by the sponsor or a representative from any other testing party (i.e., sponsor’s designee). UAT should not be considered completed until this form is signed.

Once UAT has been completed, all UAT documentation (e.g., UAT Test Plan, completed test cases, UAT Summary Report, and UAT approval form) should be archived for maintenance as essential study documents. As a final step for closure of UAT, the sponsor and/or designee should review the agreed-upon UAT performance metrics. The Metrics Champion Consortium (MCC) has a standard set of UAT metrics designed to assess the performance of the UAT [8]. It is recommended the sponsor (or designee) utilize the MCC metrics to support the evaluation of the UAT.

## Conclusion

In summary, although UAT may be performed differently among eCOA providers and sponsors, the end goal is to ensure proper documentation of UAT activities. Various techniques may be used depending on the nature of the eCOA system and the study. Rigorous and complete testing will facilitate successful system deployment, while thorough documentation of UAT will meet requirements for regulatory inspection. Completing the full UAT process using these best practices will help reduce the risk that a system does not meet the expectations of the stakeholders within a study. A thorough UAT process will also minimize the risk of inaccurate or missing data due to undetected flaws in the system that could jeopardize the results of the study and product approval. Following these best practice recommendations and completing UAT in its entirety will help support a high quality eCOA system and ensure more reliable and complete data are collected, which are essential to the success of the study.

## Glossary

Ad hoc testing: Ad hoc testing is a less formal testing method compared to following test scripts. It involves testing available functionality in a way that may not be detailed in the test script. Results from this exploratory testing may still be recorded during the UAT process
Bug tracking tool: Used by the eCOA provider to track errors in code found during testing
COA: Clinical outcome assessment. Assessment of a clinical outcome can be made through report by a clinician, a patient, a non-clinician observer, or through a performance-based assessment. Types of COAs include: Patient-reported outcome (PRO) measures, Clinician-reported outcome (ClinRO) measures, Observer-reported outcome (ObsRO) measures, Performance outcome (PerfO) measures, (Source: “BEST (Biomarkers, EndpointS, and other Tools) Resource”) [9]. In clinical trials, COAs are intended to provide evidence regarding clinical benefit (i.e., how patients feel or function in their daily lives as a result of the treatment)
CRO: Contract research organization. An organization that provides support to the pharmaceutical, biotechnology, and medical device industries in the form of research services outsourced on a contract basis
Data loading: The process of copying and loading data or data sets from a source file, folder, or application to a database or similar application (also referred to as dummy data; test dataset). Data loading is not a standard practice in all studies and may be used during UAT when there is a need to test complex patterns or an extensive date range of data by which manual data entry is not feasible
eCOA: Electronic clinical outcome assessment. A clinical outcome assessment that has been implemented on an electronic data collection platform (e.g., smartphone or tablet)
eCOA provider: An individual, institution, company, or organization that supplies the system(s) for electronic data collection within the planned study
EDC: Electronic data capture. The process of collecting clinical trial data into a permanent electronic form. NOTE: Permanent in the context of these definitions implies that any changes made to the electronic data are recorded with an audit trail
ePRO: Electronic patient-reported outcome. Patient-reported outcome data initially captured electronically. NOTE: Usually ePRO data are captured as eSource
Findings log: Used to collate all instances in which the system does not perform as expected and are identified by testers (e.g., sponsor or designee) during UAT
IRT: Interactive response technology. The technologies that research sites use to enroll patients into clinical trials, randomize patients, and manage study drug supplies. Interactive voice response system (IVRS) or interactive web response system (IWRS) falls under the IRT umbrella
ISPOR: A non-profit member-driven organization formed to promote the practice and enhance the science of health economics and outcomes research (formerly the International Society for Pharmacoeconomics and Outcomes Research).
QA: Quality assurance. All those planned and systematic actions that are established to ensure that the trial is performed, and the data are generated, documented (recorded), and reported in compliance with good clinical practice (GCP) and the applicable regulatory requirement(s) [9]
Requirements document: A document defining the business and study needs and detailing the functionality of a system developed for use in a study to meet those needs
SDLC: Software development life cycle. Process for planning, creating, testing, and deploying software. There are usually six stages in this cycle: requirement analysis, design, development and testing, implementation, documentation, and evaluation
Sponsor: In the conduct of a clinical study, a sponsor is an individual, institution, company, or organization that takes the responsibility to initiate, manage, or finance the clinical study, but may not actually conduct the investigation
SOP: Standard operating procedure. A set of step-by-step instructions compiled by an organization to help workers carry out complex routine operations. SOPs aim to achieve efficiency, quality output, and uniformity of performance, while reducing miscommunication and failure to comply with industry regulations. Detailed, written instructions to achieve uniformity of the performance of a specific function [10]
System: People, machines, software, applications, and/or methods organized to accomplish a set of specific functions or objectives [11]
System validation: The documented process of ensuring a system is functioning correctly and has no errors. This extends also to any custom system requirements that would be defined within the requirements document
UAT: User acceptance testing. The last phase of the system testing process. System users (e.g., the sponsor or its designee) use test cases and test scripts written against the requirements document to test the eCOA system to ensure it performs as expected. Any testing that takes place outside of the developer's controlled environment. [from FDA General Principles of Software Validation; Final Guidance [3], Sect. 5.2.6]. *Recognize that ‘UAT’ in other industries refers to the end user.
UI: User interface. The component of an information system with which a person may interact. This can include a display screen, stylus, keyboard, mouse, and the appearance of a desktop

## References

[1] Coons SJ, Gwaltney CJ, Hays RD, Lundy JJ, Sloan JA, Revicki DA, Lenderking WR, Cella D, Basch E (2009). Recommendations on evidence needed to support measurement equivalence between electronic and paper-based patient-reported outcome (PRO) measures: ISPOR ePRO Good Research Practices Task Force Report. Value Health.

[2] Zbrozek A, Hebert J, Gogates G, Thorell R, Dell C, Molsen E (2013). Validation of electronic systems to collect patient-reported outcome (PRO) data—recommendations for clinical trial teams: report of the ISPOR ePRO Systems Validation Good Research Practices Task Force. Value Health.

[3] US Department of Health and Human Services. Food and Drug Administration. General principles of software validation; final guidance for industry and FDA staff. 2002. https://www.fda.gov/media/73141/download. Accessed 25 Oct 2021

[4] US Department of Health and Human Services. Food and Drug Administration. Guidance for industry: computerized systems used in clinical investigations. 2007. https://www.fda.gov/media/70970/download. Accessed 12 Oct 2021

[5] US Department of Health and Human Services. Food and Drug Administration. Guidance for industry: patient-reported outcome measures: use in medical product development to support labeling claims. https://www.fda.gov/media/77832/download. Accessed 25 Oct 2021

[6] Eremenco S, Coons SJ, Paty J, Coyne K, Bennett A, McEntegart D (2014). PRO data collection in clinical trials using mixed modes: report of the ISPOR PRO Mixed Modes Good Research Practices Task Force. Value Health.

[7] US Department of Health and Human Services. Food and Drug Administration. Discussion document for patient-focused drug development public workshop on guidance 3: select, develop or modify fit-for-purpose clinical outcome assessments. 2018. https://www.fda.gov/media/116277/download. Accessed 12 Oct 2021

[8] Metrics Champion Consortium. https://metricschampion.org/. Accessed 12 Oct 2021

[9] BEST (Biomarkers, EndpointS, and other Tools) Resource. http://www.ncbi.nlm.nih.gov/books/NBK338448. Accessed 12 Oct 2021

[10] International Council on Harmonisation Good Clinical Practice. Glossary. https://ichgcp.net/1-glossary. Accessed 12 Oct 2021

[11] American National Standards Institute. http://www.ansi.org/. Accessed 12 Oct 2021

